# Dynamic Changes in the Intraepithelial Lymphocyte Numbers Following *Salmonella* Typhimurium Infection in Broiler Chickens

**DOI:** 10.3390/ani14233463

**Published:** 2024-11-30

**Authors:** Shuja Majeed, Bikas R. Shah, Nimra Khalid, Lisa Bielke, Ali Nazmi

**Affiliations:** 1 Department of Animal Sciences, College of Food Agriculture and Environmental Sciences, The Ohio State University, Wooster, OH 44691, USAkhalid.78@osu.edu (N.K.); 2 Department of Wildlife and Ecology, University of Veterinary and Animal Sciences, Lahore 54000, Punjab, Pakistan; 3 Prestage Department of Poultry Science, College of Agriculture and Life Sciences, North Carolina State University, Raleigh, NC 27695, USA; lbielke@ncsu.edu; 4 Food for Health Discovery Theme, The Ohio State University, Columbus, OH 43210, USA

**Keywords:** Salmonellosis, immune response, intraepithelial lymphocytes, gene expression, cytokines, chicken

## Abstract

At day 21 of age, Ross-308 broilers were orally gavaged with 7.5 × 10^6^ CFU/mL S. Typhimurium (n = 30), and another 30 birds were kept as the control. The body weight of birds was recorded on days 0, 2, 7, and 14 days post-infection (dpi) to calculate body weight gains (BWGs). At each time point, seven birds per group were euthanized for sample collection to acquire IELs and lymphocytes from the ileum and spleen for flow cytometric analysis. A reduction in BWGs of the infected groups compared to the control group was observed only at 2 dpi. Additionally, there were no changes in the expression of *IFN-γ*, *IL-1β*, and *TNF-α* in the ileum at 2 and 7 dpi. The number of IELs increased significantly following *Salmonella* infection in the ileum at 2 and 7 dpi without any changes in spleen lymphocytes. The increase in the total number of IELs was derived from the elevated numbers of conventional CD8αβ^+^TCRαβ^+^ and natural IEL populations (CD4^−^CD8^−^TCRαβ^+^, CD8αα^+^TCRαβ^+^, TCRγδ^+^, non-T cells (TCR^neg^, and iCD8α cells)). The increase in regulatory IELs and the stable expression of proinflammatory cytokine genes during the first week of infection suggests the potential role of IELs in modulating intestinal inflammation.

## Simple Summary

*Salmonella* is a significant foodborne disease affecting the poultry industry and human health worldwide. Understanding the protective role of the intestinal mucosal immune response to *Salmonella* infection is critical to developing immune-based strategies to control *Salmonella* infection in chickens. Therefore, this study was conducted to determine the kinetics of intraepithelial lymphocytes (IELs) and their role in *Salmonella enterica* serovar Typhimurium (S. Typhimurium) infection. The results revealed that various IEL subtypes respond to *Salmonella* infection, highlighting their essential role during infection.

## 1. Introduction

The prevalence of foodborne illnesses has drawn increasing attention to the role of bacterial pathogens, particularly *Salmonella*, in the contamination of poultry products, with chicken being a prominent concern. *Salmonella*, a Gram-negative bacterium, is recognized as a leading cause of foodborne infections globally, and its association with poultry, including chicken, presents a significant public health challenge. Salmonellosis, caused by numerous strains/serotypes of *Salmonella enterica* bacteria, manifests through disturbances in the gastrointestinal tract. In chickens, *Salmonella enterica* serovar Typhimurium (S. Typhimurium) typically induces a subclinical infection, with occasional instances of severe disease reported in young chicks [1]. An alarming concern arises from subclinical infections in chickens, which are challenging to diagnose and may contaminate poultry meat and eggs. These contaminated products can enter the human food chain, causing severe food poisoning, especially in immunocompromised, elderly, and young individuals [2]. Therefore, S. Typhimurium does not only cause economic losses in the poultry industry but also raises concerns for human health.

To some extent, the immune response to *Salmonella* has been studied in chickens, such as research by Sheela et al., where they concluded that the *Salmonella enterica* serovar Enteritidis challenge elevates gut-associated T cells, IgA, and IgG [3]. Moreover, the *Salmonella* challenge results in the upregulation of proinflammatory genes, such as *IL-1β*, *IFN-γ*, and *IL-17*, in the chicken cecum [4]. An increase in granulocytes and cytotoxic T cells (CD8αβ^+^ TCRαβ^+^) have also been observed in chicken ceca following *Salmonella enterica* infection [5]. However, there are a few studies on how intraepithelial lymphocytes (IELs) in the intestine respond to *Salmonella* infection in chickens. Therefore, this study aims mainly to characterize and quantify the IEL subset in the ileum. Understanding the characteristics of these cells could lead to developing preventive strategies against enteric infections in poultry.

IELs are interspersed within the intestinal epithelium and are considered first responders to enteric pathogens due to their proximity to the intestinal lumen [6]. Interestingly, these cells also interact with commensal microbiota and food without resorting to inflammation and cytotoxic effector functions, signifying their tolerogenic properties. IELs have been broadly researched in humans and mice, and the majority consist of T lymphocyte subtypes. They are broadly classified into induced/conventional and natural/unconventional IELs [7]. Induced IELs encompass classical lymphocytes: T helper cells (CD4^+^TCRαβ^+^) and cytotoxic T cells (CD8αβ^+^TCRαβ^+^) are initially activated in the periphery and, upon encountering their specific antigen, migrate to the IEL compartment. Natural IELs, on the other hand, migrate to the intestinal epithelium as soon as they mature in the thymus and consist of T cells (TCRγδ^+^, CD8αα^+^TCRαβ^+^, and CD4^−^CD8^−^TCRαβ). Non-T cell IELs are part of natural IELs and constitute the innate lymphoid cell (ILC)-1, ILC-3, intracellular CD3 (iCD3^+^), and innate CD8α (iCD8α^+^) [7].

IEL research is limited to chickens, although some characterization studies have been conducted. In 1988, Lawn and colleagues documented the presence of IELs in chickens, noting that the majority of chicken IELs resemble T cell IELs found in humans and mice [8]. Later, Meijerink et al. concluded that NK cells also comprise a portion of the chicken IEL population [9]. Some studies have investigated how IELs respond to specific pathogens. For instance, one study reported changes in the IEL population and alterations in proinflammatory cytokine gene expressions following an *Eimeria* challenge [10]. Additionally, differences in the IEL population were observed during viral infection when chickens were challenged with the Newcastle disease virus [11]. However, this research has primarily focused on TCRγδ^+^, CD4^+^, and CD8^+^ T cells without exploring other subsets within IELs. Our laboratory was the first to characterize numerous IEL subsets in chickens in response to *Clostridium perfringes* infection. We have reported that natural IEL subsets (TCRαβ^+^CD4^−^CD8^−^, TCRαβ^+^CD8αα^+^, TCRγδ^+^, TCR^neg^, and iCD8α^+^) exhibit elevated numbers during early stages of necrotic Enteritis in chickens [12]. However, a detailed analysis of the IEL population response to *Salmonella* has yet to be investigated. Therefore, in the present study, we infected the chickens with S. Typhimurium to determine the kinetics of IELs during the course of infection. Furthermore, we evaluated the gene expression of proinflammatory cytokines in the ileum to determine intestinal inflammation during Salmonellosis in poultry.

## 2. Materials and Methods

### 2.1. Bacterial Culture

The S. Typhimurium strain (the poultry isolate obtained by USDA National Veterinary Services Lab, Ames, IA, USA), graciously provided by Dr. Lisa Bielke [13], was propagated in LB broth (Sigma-Aldrich, St. Louis, MO, USA), followed by stock solution preparation in sterile phosphate-buffer saline (PBS). The concentration was adjusted to 1 × 10^7^ CFU/mL utilizing a spectrophotometer at a 625 nm wavelength. Subsequently, the concentration was validated by plating on XLT-4 agar (Sigma-Aldrich, USA), confirming the bacterial dose to be 7.5 × 10^6^ CFU/mL.

### 2.2. Husbandry, Experimental Design, and Performance

Sixty Ross 308 broiler chicks were housed in a floor pen covered with wood shaving in an environment-controlled housing facility designated as BSL-2. At 21 days of age (0 days post-infection (dpi)), the birds were divided into two groups (control and S. Typhimurium), weighed individually, and placed in two separate floor pens, each with thirty birds. The control group was administered 1 mL PBS, while the S. Typhimurium group was orally gavaged with 7.5 × 10^6^ CFU/mL of S. Typhimurium per bird (Supplementary Figure S1). At 2, 7, and 14 dpi, individual birds were weighed to calculate body weight gains (0–2 dpi, 0–7 dpi, and 0–14 dpi BWGs). At each time point, 7 chickens from each group were randomly selected and harvested for sample collections. Throughout the duration of the trial, mortality was recorded, and birds were observed for any clinical signs following the infection. The ileum and spleen were sampled for the isolation of IELs and T lymphocytes, respectively, and an additional ileum section was obtained for RNA isolation. Additionally, the cecal contents and spleen were collected to indicate local colonization levels and systematic infection, respectively.

### 2.3. Salmonella Recovery and Count

Ceca contents and spleen were collected in PBS and placed on ice. Tissues were weighed, homogenized, serially diluted in PBS, and plated on XLT-4 agar plates (Sigma-Aldrich, USA) and incubated at 37 °C for 24 h. Bacterial colonies were counted to obtain CFU/g and assess the recovery of S. Typhimurium from the birds. In the case of the absence of colonies, the samples were enriched overnight in tetrathionate broth at 37 °C. Following enrichment, the samples were re-plated on XLT-4 agar and incubated at 37 °C for 24 h.

### 2.4. Isolation of Immune Cells and Flow Cytometry

The isolation of IELs and flow cytometry were performed according to an established mouse protocol [14], which was modified for chickens [12]. At 2, 7, and 14 dpi, an approximately 7 cm segment of the middle ileum was removed, flushed with PBS, opened longitudinally to remove any feces and mucus, weighed, and cut into smaller pieces (approx. 1 cm). The ileum fragments were collected in a 50 mL tube containing PBS supplemented with 5% chicken serum (Sigma-Aldrich, USA), 2 mM EDTA (Quality Biological, Gaithersburg, MD, USA), and 2 mM dithiothreitol (Sigma-Aldrich, USA). Afterward, the tubes were placed in an incubator shaker (37 °C, 150 rpm) for 45 min. The supernatant was passed through the gauze column resuspended in 40% and overlaid on a 70% Percoll (Cytiva, Marlborough, MA, USA) density gradient to acquire the IEL fraction. To isolate splenocytes, the spleen tissues were crushed with a syringe plunger on a 70 µL cell strainer and rinsed in a supplemented PBS medium. Recovered IELs and splenocytes were incubated with an ACK buffer (Quality Biological, USA) for 5 min to lyse red blood cells. Then, cells were washed and resuspended in a staining buffer. Live cells were quantified using the Trypan blue exclusion method [15]. An aliquot of cells (1 × 10^6^) was stained with fluorochrome-conjugated anti-chicken CD45 SPRD (LT40), CD4 PE-CY7 (CT-4), CD3 AF547 (CT-3), TCRγδ FITC (TCR-1), CD8α AF700 (CT-8), and CD8β PE (EP42) antibodies (Southern Biotech, Birmingham, AL, USA). Ghost viability dye-Red 510 (Tonbo Biosciences, San Diego, CA, USA) was used to distinguish between live and dead cells. Stained cells were acquired through BD FACSCanto II flow cytometry (BD Biosciences, Franklin Lakes, NJ, USA). FlowJo v10.8.1 software (BD Biosciences, USA) was used to analyze the frequency of cells, and the gating strategy used was similar to our previous study [12]. The quantity of cells was expressed as the number of cells per gram of tissue.

### 2.5. RNA Isolation and Gene Expression

The ileum tissue section was collected in RNA later (Thermo Fisher Scientific, Waltham, MA, USA). The Monarch^®^ Total RNA Miniprep Kit (New England Biolabs^®^, Ipswich, MA, USA) was used to isolate RNA from the ileum, and cDNA was synthesized from the isolated RNA using the LunaScript^®^ RT SuperMix Kit (New England Biolabs^®^, USA). Luna^®^ Universal qPCR Master Mix (New England Biolabs^®^, USA) was used to run real-time PCR in a Bio-Rad CFX Connect machine (Bio-Rad Laboratories, Hercules, CA, USA). The primer sequences for *IFN-γ*, *IL-1β*, and *TNF-α* genes are displayed in Table 1. The cycle threshold (Ct) for each gene was normalized to the housekeeping gene, *GAPDH*. Relative fold change was calculated compared to the control group using the 2^−ΔΔCt^ method [16].

### 2.6. Statistical Analysis

GraphPad PRISM v10.0.03 (GraphPad, Boston, MA, USA) was employed for data analysis, and individual chickens were considered an experimental unit. The Mann–Whitney U test was utilized to analyze flow cytometry data, and Student’s *t*-test was used for BWGs, bacterial count, and gene expression. *p* < 0.05 was considered statistically significant.

## 3. Results

### 3.1. Chicken Performance

**Figure 1:**
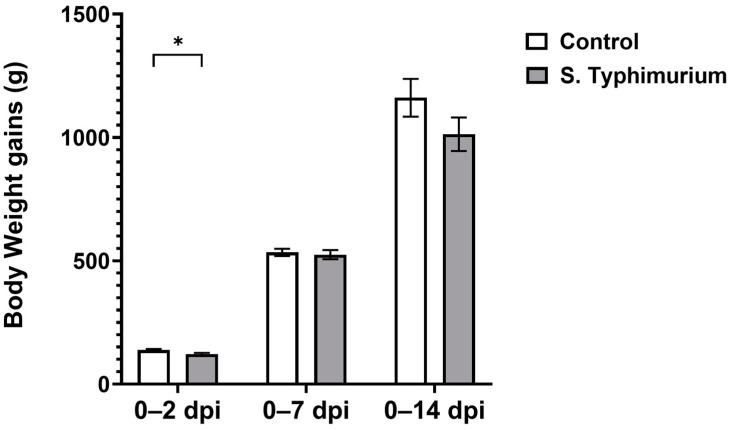
Body weight gain following the *Salmonella* challenge. The control group was administered PBS, while the S. Typhimurium group was challenged with 7.5 × 10^6^ CFU/mL of S. Typhimurium at 21 days of age (0 dpi). Bars represent the mean values (n = 30 for 0–2 dpi, 23 for 0–7 dpi, 16 for 0–14 dpi), and the error lines indicate SEM. * *p* < 0.05.

BWGs decreased significantly (*p* < 0.05) at 2 dpi for the S. Typhimurium group, but they recovered by 7 dpi and were comparable to the control group at 14 dpi (Figure 1). No mortality or clinical signs were observed during the course of infection.

### 3.2. Salmonella Loads

*Salmonella* loads in cecal contents were examined to confirm infection. The results verified that the infected group harbored S. Typhimurium, and their bacteria load decreased over time (3.44 × 10^8^ CFU/gram at 2 dpi, 5.63 × 10^2^ CFU/gram at 7 dpi, and 3.13 × 10^1^ CFU/gram at 14 dpi). No bacteria were detected in the control group. Spleen samples of the S. Typhimurium group showed that 47% and 73% of birds were *Salmonella*-positive at 2 and 7 dpi, respectively.

### 3.3. Effect of S. Typhimurium Challenge on IEL Populations

**Figure 2:**
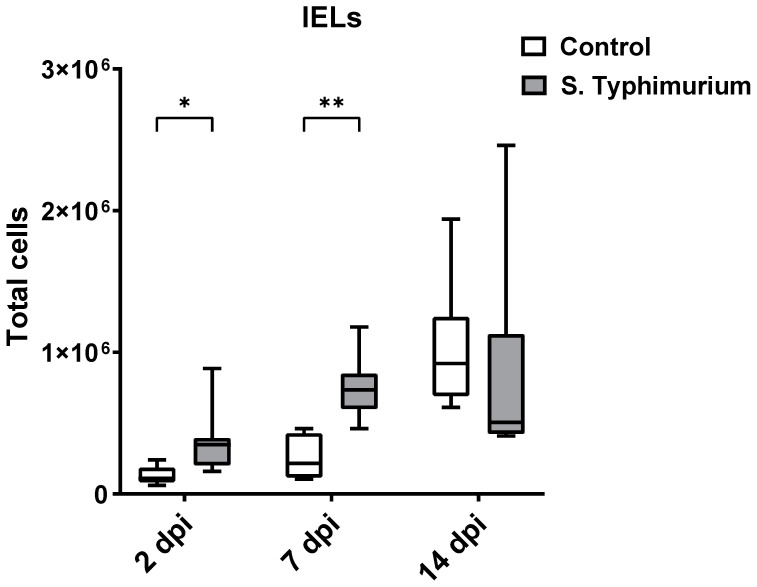
Effect of the *Salmonella* challenge on IELs number (cells/gram of tissue) in the ileum. The control group was administered PBS, while the S. Typhimurium group was challenged with 7.5 × 10^6^ CFU/mL of S. Typhimurium at 21 days of age (0 dpi). The box plot shows the distribution of the data, and the central line shows the median (n = 7), while whiskers depict variability outside the upper and lower quartiles. * *p* < 0.05, ** *p* < 0.01.

**Figure 3:**
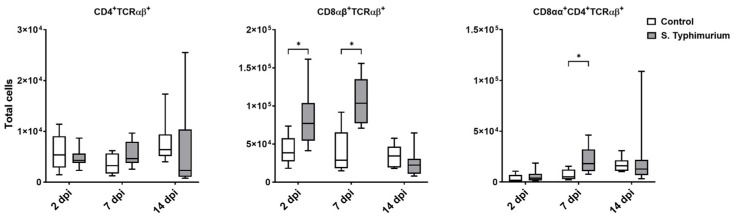
Conventional IEL subpopulation (cells/gram of tissue) in the ileum following the *Salmonella* challenge. The control group was administered PBS, while the S. Typhimurium group was challenged with 7.5 × 10^6^ CFU/mL of S. Typhimurium at 21 days of age (0 dpi). The box plot shows the distribution of the data, and the central line shows the median (n = 7), while whiskers depict variability outside the upper and lower quartiles. * *p* < 0.05.

**Figure 4:**
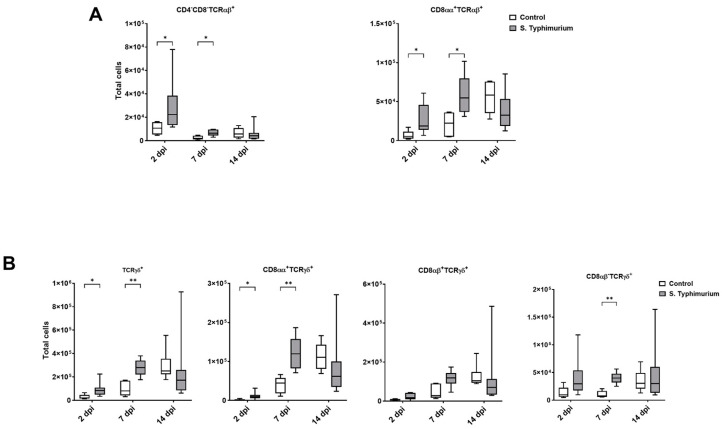
Natural IEL population (cells/gram of tissue) in the ileum following the *Salmonella* challenge. (**A**) TCRαβ natural IELs; (**B**) TCRγδ natural IELs. The control group was administered PBS, while the S. Typhimurium group was challenged with 7.5 × 10^6^ CFU/mL of S. Typhimurium at 21 days of age (0 dpi). The box plot shows the distribution of the data, and the central line shows the median (n = 7), while whiskers depict variability outside the upper and lower quartiles. * *p* < 0.05; ** *p* < 0.01.

**Figure 5:**
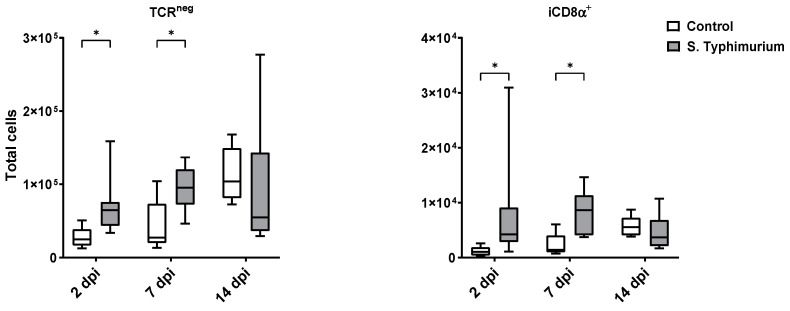
The number of TCR^neg^ cells and their subpopulation (cells/gram of tissue) in ileum after the *Salmonella* infection. The control group was administered PBS, while the S. Typhimurium group was challenged with 7.5 × 10^6^ CFU/mL of S. Typhimurium at 21 days of age (0 dpi). The box plot shows the distribution of the data, and the central line shows the median (n = 7), while whiskers depict variability outside the upper and lower quartiles. * *p* < 0.05.

Through flow cytometry, we acquired diverse IEL subsets to study their response to S. Typhimurium infection. We focused on the ileum since it is one of the primary locations for *Salmonella* colonization in chickens [19]. Our results showed that the number of IELs increased significantly following *Salmonella* infection in the ileum at 2 and 7 dpi (Figure 2). Despite the numerical increases in IELs at 14 dpi, both infected and control groups had comparable numbers of IELs. Then, we performed a deep analysis of the IEL subpopulation by flow cytometry. Most of the increase in the number of total IELs was derived from the elevated numbers of conventional CD8αβ^+^TCRαβ^+^ and natural IEL populations (CD4^−^CD8^−^TCRαβ^+^, CD8αα^+^TCRαβ^+^, TCRγδ^+^, and non-T cells (TCR^neg^) (Figure 3, Figure 4 and Figure 5). Furthermore, there was a significant increase in the number of CD8αα^+^CD4^+^TCRαβ^+^ cells in the *Salmonella*-infected birds at 7 dpi only (Figure 3, right panel). Chicken TCRγδ T cells are well-known for expressing both CD8αα homodimers and CD8αβ heterodimers on the cell surface [20]. Therefore, we further characterized TCRγδ IELs according to their expression of CD8α and/or CD8β. The ileum of the S. Typhimurium group harbored significant increases in the number of CD8αα^+^TCRγδ^+^ cells at 2 and 7 dpi, as well as CD8αβ^-^TCRγδ^+^ cells at 7 dpi (Figure 4B). However, the number of CD8αβ^+^TCRγδ^+^ cells was comparable between groups during the course of infection. Finally, the number of iCD8α cells, which represent approximately 10% of TCR^neg^ IELs, displayed a two-fold increase in the S. Typhimurium group compared to the control group at 2 and 7 dpi (Figure 5, left panel).

### 3.4. Spleen Lymphocytes

**Figure 6:**
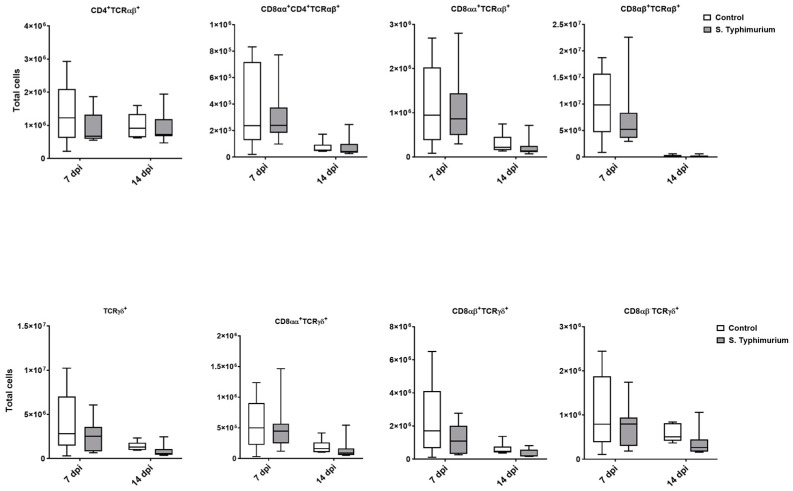
Spleen lymphocyte (cells/gram of tissue) number following the *Salmonella* challenge. The control group was administered PBS, while the S. Typhimurium group was challenged with 7.5 × 10^6^ CFU/mL of S. Typhimurium at 21 days of age (0 dpi). The box plot shows the distribution of the data, and the central line shows the median (n = 7), while whiskers depict variability outside the upper and lower quartiles.

We performed flow cytometry analysis on splenic lymphocytes, especially focusing on T cells at 2, 7, and 14 dpi. However, due to a technical issue with the flow cytometry machine during acquisition, the 2 dpi samples were lost. Surprisingly, no changes in the T cell populations were observed between groups (Figure 6).

### 3.5. Gene Expression of Proinflammatory Cytokines

**Figure 7:**
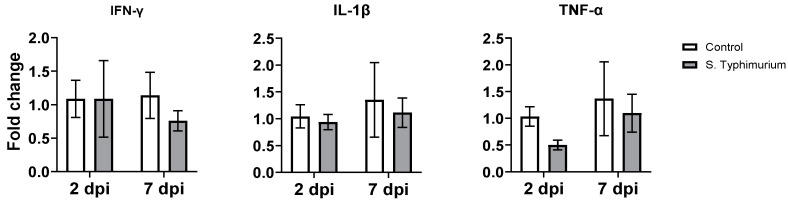
The effect of *Salmonella* on the gene expression of selected genes in the ileum. The control group was administered PBS, while the S. Typhimurium group was challenged with 7.5 × 10^6^ CFU/mL of S. Typhimurium at 21 days of age (0 dpi). Bars represent the mean values (n = 4), and the error lines indicate SEM.

There were no changes in the gene expression of proinflammatory cytokines (*IFN-γ*, *IL-1β*, and *TNF-α*) in the ileum of both the infected and control groups (Figure 7).

## 4. Discussion

Our study shows that *Salmonella*-infected birds exhibited no clinical signs with no mortality. There were no differences in body weight compared to the control birds, as BWGs remained similar between groups throughout the infection except for 2 dpi. Numerous other studies have also concluded that birds shed S. Typhimurium without displaying any overt clinical signs and symptoms [21,22,23,24,25].

Research on T cells has confirmed their vital role in *Salmonella* defense response [26]. Henceforth, we focused on IELs, primarily comprising T cells. At 2 and 7 dpi, there were significant increases in the number of IELs in the ileum, while the spleen lymphocytes remained unchanged. Broiler chicken’s early infection with *Salmonella enterica* serotype Enteritidis induced IEL infiltration in the ileum as early as 1 dpi, without any changes in splenic leukocytes [27]. Furthermore, the number of ileum cytotoxic CD8 T IELs was elevated during the first week of infection [27]. The later result aligned with ours, showing remarkable increases in conventional CD8αβ^+^TCRαβ^+^ cells in the infected group at 2 and 7 dpi, highlighting the importance of these cells during Salmonellosis. The cytotoxic CD8 T cells are an essential arm of adaptive immunity, primarily responsible for eliminating intracellular pathogens and cancerous cells [28]. In mice, cytotoxic CD8 T cells are critical to accelerating S. Typhimurium clearance [29], particularly during the late stage of infection with attenuated *Salmonella* [30]. The cytotoxic CD8 T cells displayed elevated levels of IFN-γ production, potentially enhancing the killing of *Salmonella*-infected macrophages [29].

Our results demonstrated that the ileum of the infected group harbored significantly higher numbers of two regulatory cell types: CD8αα^+^CD4^+^TCRαβ^+^ IELs at 7 dpi and CD8αα^+^TCRαβ^+^ IELs at 2 and 7 dpi. This might explain the absence of clinical signs in the S. Typhimurium-infected group, which coincided with similar expression levels of proinflammatory cytokine genes (*IFN-γ*, *IL-1β*, and *TNF-α*) between groups. Sadeyen et al. [31] investigated two chicken lines for proinflammatory cytokine expression and reported no difference from the control in *IFN-γ* expression in both lines and a similar IL-1β expression in one line during early *Salmonella* infection. A study of the suppressive potency of Foxp3^+^T regulatory cells during persistent *Salmonella* infection in mice indicates the presence of these cells, progressively increasing bacterial burden by delaying the activation of effector T cells [32]. Similarly, during chicken infection with *Salmonella* Enteritidis, the number of CD4^+^CD25^+^ T regulatory cells significantly increased with the up regulation of the anti-inflammatory cytokine IL-10 in the cecal tonsils of infected birds [33].

CD8αα^+^CD4^+^TCRαβ^+^ and CD8αα^+^TCRαβ^+^ IEL development and functions are well studied in mice. In chickens, however, only one study by our group indicated the importance of CD8αα^+^TCRαβ^+^, but not CD8αα^+^CD4^+^TCRαβ^+^ cells during necrotic Enteritis infection [12]. In mammals, CD8αα^+^CD4^+^TCRαβ^+^ IELs are generated from peripheral CD4^+^ TCRαβ^+^ cells when they migrate into the intestinal epithelium, where they acquire CD8αα expression [6,34]. There is also strong evidence that lamina propria regulatory T cells are precursors to CD8αα^+^CD4^+^TCRαβ^+^ cells, but they lose Foxp3 expression [35]. The expression of CD8αα requires the upregulation of transcription factors (Runx3 and T-bet) by CD4 T cells [36,37,38], epithelial signals (retinoic acid, IFN-γ, and IL-27) [37,38,39,40], and the intestinal microbiome, particularly *Lactobacillus reuteri* [41]. Studies in humans with inflammatory bowel diseases suggested the anti-inflammatory function of CD8αα^+^CD4^+^TCRαβ^+^ IELs [42,43]. In addition, these cells protected immunodeficient mice against intestinal colitis mediated by IL-10 [44]. CD8αα^+^TCRαβ^+^ cells are uniquely present in the IEL compartment, where they are generated from thymic emigrant CD4^−^CD8^−^TCRαβ^+^ cells by gaining CD8αα expression in the intestinal epithelium [45,46,47,48]. The development and maintenance of CD8αα^+^TCRαβ^+^ IELs require TGF-β, IFN-γ, IL-15, the Aryl hydrocarbon receptor, and microbiota, as well as Runx3 and T-bet [49]. CD8αα^+^TCRαβ^+^ IELs express immunomodulatory factors such as TGF-β3, lymphocyte activation 3, and fibrinogen-like protein 3 [50]. Moreover, they prevent the development of colitis after the adoptive transfer of naïve CD4 T cells into immunodeficient mice [51,52]. Together, the elevation of the number of IEL populations with regulatory functions during the first week of infection may explain the immune tolerance of chickens against *Salmonella* colonization and other commensal organisms [53,54,55,56,57]. However, more studies on chicken regulatory IELs are required to confirm this conclusion.

TCRγδ^+^ cells rapidly increase in various animal species following *Salmonella* infection [58,59,60]. TCRγδ^+^ cells, which represent 50% of total T cells in the IEL compartment, produce anti-inflammatory cytokines (TGF-β and IL-10), proinflammatory cytokines (TNF-α and IFN-γ), antimicrobial proteins, wound healing factors, and cytotoxicity proteins such as granzymes, among others [7]. In chickens, there was an expansion of the circulating CD8αα^+high^TCRγδ^+^ cells in the blood following oral vaccination with *Salmonella enterica* serovar Enteritidis [58]. In addition, the frequency of TCRγδ^+^ and CD8αα^+^TCRγδ^+^ cells in the peripheral blood, spleen, and cecum increased at 4 and 7 dpi of S. Typhimurium infection in young chicks [61]. Consistent with these findings, our data underscored the crucial role of TCRγδ^+^ IELs in anti-*Salmonella* mucosal immunity, demonstrated by the significant expansion of these cells and CD8αα^+^TCRγδ^+^ IELs in the ileum of infected birds at 2 and 7 dpi.

In mice and humans, TCR^neg^ IELs are composed of innate-like lymphoid cells expressing NKP46, NK1.1, and/or NKP44 receptors [62,63,64], as well as lymphocytes expressing intracellular CD3γ (iCD3γ cells) that express CDαα (iCD8α cells) [8,65]. In chickens, only two subsets of the TCR^neg^ IELs population have been characterized: natural killer (NK) cells [9,66] and iCD8α^+^ cells [12]. Previous studies have indicated their involvement in chicken intestinal responses to enteric diseases. For instance, the number of natural NK IELs peaked at 1 dpi following infection with *Salmonella enterica* serovar Enteritidis and gradually decreased over time [27]. The iCD8α^+^ cells increased numerically during necrotic Enteritis in chickens at 1 dpi [12]. In our current study, the S. Typhimurium group presented significant increases in the number of non-T cell IELs, TCR^neg^ cells, and their subset iCD8α^+^ cells in the ileum at 2 and 7 dpi, suggesting the pivotal role of IELs with innate functions during early *Salmonella* infection.

## 5. Conclusions

We demonstrated that the diverse IEL population in the ileum of broilers responds to S. Typhimurium during the first week of infection. These IELs predominantly function as cytotoxic, regulatory, innate-like, and/or innate cells within the immune system. The increase in these IEL populations in the *Salmonella*-infected group coincided with a reduction in the cecal *Salmonella* load. Enhancing our understanding of IEL functions will be crucial for comprehending the host immune response to *Salmonella* and for developing effective preventive strategies against enteric diseases in chickens. Therefore, we plan to sort IEL subsets, particularly CD8αα^+^CD4^+^TCRαβ^+^ and CD8αα^+^TCRαβ^+^ IEL, and perform single-cell sequencing to precisely delineate their exact functions.

## Figures and Tables

**Table 1:** List of primer sequences used for quantitative real-time PCR.

Gene	Accession No.	Primer Sequence		Product Length (bp)
		Forward (5′-3′)	Reverse (5′-3′)	
*IL-1β* [17]	XM_015297469.1	CCCGCCTTCCGCTACA	CACGAAGCACTTCTGGTTGATG	66
*IFN-γ* [17]	NM_205149.1	GCTCCCGATGAACGACTTGA	TGTAAGATGCTGAAGAGTTCATTCG	63
*TNF-α* [18]	MF000729.1	CCCATCCCTGGTCCGTAAC	ATACGAAGTAAAGGCCGTCCC	77
*GAPDH* [18]	NM_204305	CCTAGGATACACAGAGGACCAGGTT	GGTGGAGGAATGGCTGTCA	64

*IL-1β*, *interleukin 1beta*; *IFN-γ*, *interferon-gamma*; *TNF-α*, *tumor necrosis factor alpha*; *GAPDH*, *glyceraldehyde 3-phosphate dehydrogenase*.

## Data Availability

Data are available on request.

## Supplementary Materials

The following supporting information can be downloaded at: https://www.mdpi.com/article/10.3390/ani14233463/s1, Supplementary Figure S1: Schematic of experimental design.
