# Supplementary material for: Dynamic Changes in the Intraepithelial Lymphocyte Numbers Following Salmonella Typhimurium Infection in Broiler Chickens
Source: Animals (Basel). 2024 Nov 30;14(23):3463. doi: 10.3390/ani14233463 (PMC11640588; doi:10.3390/ani14233463)
Supplement: Supplementary file 1 [file animals-14-03463-s001.zip › animals-3294147-supplementary.pdf]

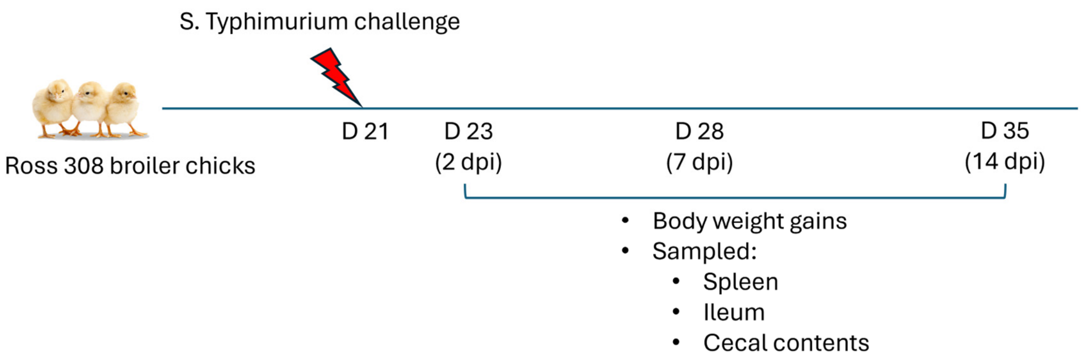

Supplementary Figure S1. Schematic of experimental design. On day 21 of age, the control group (n=30) was administered PBS, while the S. Typhimurium group (n=30) was challenged with 7.5 x 10^6 CFU/ml of S. Typhimurium. D, day of age; dpi, day post-infection.
